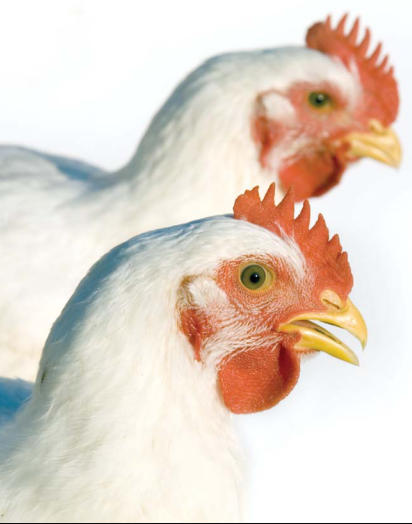# Resisting Arrest: Drug-Resistant *Campylobacter* Persists in Poultry

**Published:** 2007-07

**Authors:** Angela Spivey

Doctors use the fluoroquinolone class of antibiotics to treat food poisoning caused by *Campylobacter*. But after poultry farmers began using fluoroquinolones to treat respiratory disease in flocks, the drugs became less effective in people. In 2005, the FDA banned the use of fluoroquinolones on poultry farms because of these concerns. A new study now suggests that the ban may not be enough to fix the problem **[*EHP* 115:1035–1039; Price et al.]**.

A team of Johns Hopkins researchers made weekly trips to Baltimore supermarkets for 20 weeks in 2004 and 15 weeks in 2006. Each week, they bought chicken from each of five different producers. Three producers had never used any antibiotics; two were major conventional producers that declared they had ceased all use of fluoroquinolones in 2002, three years before the FDA ban. The scientists tested one piece of chicken from each package for *Campylobacter*, confirmed the bacterium’s identity using DNA analysis, then tested for antibiotic resistance using the minimal inhibitory concentration method.

The bacteria from conventional chicken were more likely to be fluoroquinolone-resistant than those from antibiotic-free products. The researchers compared each poultry producer to every other producer in a pair-wise fashion. In both 2004 and 2006, this statistical analysis showed that the *Campylobacter* strains from the conventionally produced chicken were more likely to be resistant than the strains from antibiotic-free samples.

In addition, between 2004 and 2006, the proportion of antibiotic-resistant bacteria on the conventionally produced chicken showed no significant change, indicating that the prevalence of fluoroquinolone-resistant *Campylobacter* was not decreasing in chicken from these producers, even after four years.

The results suggest that once antibiotic-resistant bacteria have developed, they may show up on grocery store shelves long after drug use stops. The authors note that they could not verify claims of voluntary fluoroquinolone prohibition because poultry producers are not required to report their use of drugs in food animals to regulatory agencies.

Other studies have shown that resistant bacteria can linger in poultry farms’ water distribution and ventilation systems and in reused litter. The authors state that additional interventions, such as requiring thorough disinfection and regular litter changing in poultry houses, may be necessary to reduce the public health burden of fluoroquinolone-resistant *Campylobacter*.

## Figures and Tables

**Figure:**